# Contribution of community champions to accelerate the uptake of COVID-19 vaccination in Rukwa region, Tanzania, February - October 2022

**DOI:** 10.11604/pamj.supp.2023.45.1.39705

**Published:** 2023-06-08

**Authors:** Erick Msunyaro, Jaliath Rangi, Tumaini Haonga, Neema Kileo, Jerry Mlembwa, Susan Nyawade, Zorodzai Machekanyanga, Akili Kalinga, Winfrida John, May Abdul Bukuku, Priscilla Kusena, Ibrahim Isack, Violet Mathenge, Grace Saguti, Elibahati Akyoo, Zabulon Yoti

**Affiliations:** 1 Health Promotion Section, Ministry of Health, Dodoma, Tanzania,; 2 World Health Organization, Dar es Salaam, Tanzania,; 3 World Health Organization, The Office of Regional Office, Brazzaville, Congo,; 4 National Institute for Medical Research (NIMR), Headquarters, Dar es Salaam, Tanzania,; 5 The United Nations Children’s Fund (UNICEF), Tanzania,; 6 The Office of Regional Commissioner, Rukwa, Tanzania

**Keywords:** COVID-19, vaccination, community champions, vaccine coverage, community-based campaign, Tanzania

## Abstract

**Introduction:**

Tanzania is among the African countries which started COVID-19 vaccination late (August 2021) compared to other countries in the African continent. By mid-March 2022, overall vaccine uptake was 13% of the targeted population, which was very low compared to other countries. We describe the contribution of champions in the Rukwa region that led to an increase in vaccine coverage rate in Tanzania.

**Methods:**

a community-based campaign was conducted using community champions from July 15th to August 31st, 2022. A baseline assessment was conducted focusing on the key drivers, barriers, and enablers for COVID-19 vaccine uptake in the region. A working session to develop IEC materials and messages tailored to addressing the issues raised in the community to be used in the campaign in the region was conducted, followed by the campaign’s launch. Community engagement and sensitizations, which contributed to the rise of vaccinated people, were based on house-to-house visits, village meetings, and visiting community gatherings such as marketplaces, places of worship, and sports areas, which were done by champions alongside vaccinators.

**Results:**

the campaign contributed to the increase of vaccination coverage because, before the start of the campaign, the vaccination coverage was 10% as of July 14th, 2022. After the campaign, which started on July 15th to August 31st, 2022, the coverage increased by 12%. During the post-evaluation exercise after the campaign, the coverage keeps increased and reached 94% by October 2022. The coverage kept increasing even after the intervention’s end due to the campaign’s positive effect.

**Conclusion:**

the community still needs correct information to avoid misinformation and hesitancy, especially when introducing new disease response mechanisms such as vaccines. The community champions who are based in the community play a critical role in addressing community concerns and contribute to the effectiveness of the implementation; hence sustainability is crucial.

## Introduction

Tanzania is among the African countries which started COVID-19 vaccination late compared to other countries in the African continent. In July 2021, Tanzania received the first vaccine doses donated by the US government and started vaccinating its people [1]. By mid-March 2022, overall vaccine uptake was 13% of the targeted population, which was very low compared to other countries [2-7]. The country’s low vaccination rate is mainly due to rumors, misinformation, and myths about COVID-19 vaccines. The community from both rural and urban areas had different perspectives towards vaccination [8-12]. To support the increase in vaccination rate in the country, the World Health Organization (WHO) Tanzania and the Ministry of Health mainland implemented a project of a vaccination champion. This intervention was conducted from February 2022 to the end of October 2022 by equipping and deploying community champions to motivate, inspire, and influence the target audience to get vaccinated [2,13,14].

The WHO Regional Office proposed this intervention as a pre-test to see if the champion initiative can increase vaccination coverage. Before the implementation of the campaign, a baseline assessment was conducted to study reasons for hesitance to inform the project methodologies. The study results showed that vaccine safety was among the community’s concerns, leading to vaccine hesitancy. Using vaccinated champions who became living testimony during community engagement activities was selected [3,5,14]. Another method was using religious and high-level political leaders as champions due to the community’s trust, which increased vaccination rates [8,9,11]. This paper will discuss the effectiveness of the champion campaign approach and methods through the campaign results, which are divided into phases between July 15th when the campaign was launched to August 31st, and September 1st to October 30th, 2022.

## Methods

**Approach:** the approach used in this project was designed and suggested by World Health Organization Africa Regional Office (WHO AFRO) as a pre-test to be implemented in three low-performing African countries, Burkina Faso, Sierra Leone, and Tanzania. The campaign was designed to use community champions based in the communities to motivate, inspire, and influence target audiences to vaccinate. This strategy was implemented by adopting the idea and contextualizing it to fit our country’s physical and geographical characteristics [12].

**Establishment of a task force to oversee the campaign:** the task force was established to plan and oversee project interventions in February 2022. The task force was responsible for developing criteria for champion selection and planning and monitoring all activities for the intervention. The task force comprised teams from the Central level, which were: the Ministry of Health (i.e., Health Promotion section, Immunization, and Vaccines Development Program), the President Officer Regional Administrative and Local Government, WHO Tanzania, United Nations Children´s Fund (UNICEF), and the Regional Immunization and Vaccination Officer/Regional Health Promotion Coordinator from the Rukwa region.

**Selection of intervention area:** the intervention area was selected based on low performance on the COVID-19 vaccination rate. Rukwa was among those performing poorly (6%) by the early week of March 2022. Another criterion was its similarity in terms of the setting, cultural and traditional aspects, like the Ruvuma region, which was the leading region in vaccination rate (32%) [13]. Two councils were selected (Nkasi and Sumbawanga MC) for the interventions.

**Baseline assessment:** a baseline assessment focused on the region’s key drivers, barriers, and enablers for COVID-19 vaccine uptake [2]. The rapid exercise studied and explored community behavior and evidence-based barrier analysis and seeks to document desired behavior across the spectrum of COVID-19 vaccine acceptance and uptake outcomes in the study region. The assessment was able to look deep into the grassroots and community levels. A team from Muhimbili University of Health and Allied Sciences (MUHAS), the National Institute of Medical Research (NIMR), the Ministry of Health (MOH), and the President´s Office, Regional Administrative and Local Government (PORALG) conducted the baseline. Data were collected using a structured questionnaire customized using a mobile digital tool (DHIS App version: v2.6.2) and directly transferred to the centralized server Health Promotion Section, Monitoring and Evaluation (HPS M&E) Web-Based System). Qualitative data was collected using developed and pre-tested interview and focus group guides. Data collected were analyzed using STATA 13.1. Descriptive statistics were used to summarise socio-demographic characteristics in the frequency tables with the proportions.

**Preparation of IEC/SBC materials:** the results from the baseline assessment have contributed to a message development session. Messages developed aimed at responding to hesitancy from the community managing rumors and misinformation, targeting the audience in the Rukwa region with appropriate content for different population segments eligible for the COVID-19 vaccine. A workshop was conducted to prepare Information Education and Communication (IEC) materials addressing the issues raised in the community to be used in the campaign in the region.

**Pay-per performance strategy:** during the implementation, the task force decided when engaging community champions, they should be paid according to the pay-per-performance strategy implemented by MoH and PORALG to the other performing region. After 4 weeks of implementation, a total of 100,000 Tanzanian shillings (43 USD) was agreed to be paid as the allowance. Each champion reached 250 people who will accept to be vaccinated. This method has motivated champions to conduct community engagement activities and reach above the target.

**Launching of the campaign and orientation of champions:** launching was conducted with the presence of high-level government, ministry officials as well as regional officials. The presence of high-level officials has assisted in political commitment toward vaccination. The orientation on the objectives, modality of work, and deliverables were conducted to community champions, media personnel, and coordinators who served as supervisors. The champions were provided with the working tools for the campaign, including IEC materials, job aids, and other related materials.

**Community engagement activities conducted by champions:** community champions paid visits to the families of those not vaccinated and provided awareness education on the benefits and risks of not being vaccinated. Champions visited houses with vaccinators, and vaccination was done immediately after the awareness sessions. This approach is similar to door-to-door, which proved effective in health care services [11]. Also, Village meetings and gatherings were a good opportunity for the champions to sensitize their community. These meetings were scheduled at the village levels and organized by village leaders. Champions were invited or requested sessions and provided awareness sessions on the COVID-19 vaccine. Community gatherings such as the Farmer´s day, locally named “nane nane” markets events, sports events, local beer clubs, farmers markets, the Tanzania Social Action Fund (TASAF) meetings, and weekend auctions (referred to as gulio). Champions also provided COVID-19 awareness to the boda-boda stands and groups. Champions conducted awareness sessions on COVID-19 vaccine benefits and why people should vaccinate in places of worship on Saturdays and Sundays. For Friday prayer, faith leader´s champions had an opportunity to also talk about the COVID-19 vaccines during prayer sessions. For other mosques where champions are not Muslim faith leaders, champions had a chance to provide messages to the leaders for the awareness messages to be shared. Champions paid visits to the health facilities for awareness sessions to the clients who visited the Outpatient Department (OPD) and Reproductive and Child Health (RCH) clinics. At the RCH champions were able to educate women who brought their children for vaccination that the value of the COVID-19 vaccine is the same as those of children, and women were convinced and accepted to be vaccinated.

**Validation of data and champion community activities:** a team of experts from the MoH, PORALG, WHO country office Risk Communication and Community Engagement (RCCE), Regional Health Management Team (RHMT), and Council Health Management Team (CHMT) conducted data validation activity at the health facilities, local authorities, and community champions to see if the targets were met and community engagement activities were done.

**Ethical consideration:** this campaign was done following all procedures for engaging the community. People engaged during baseline assessment signed consent forms and other formalities. The approval was done by both ministries (Ministry of Health and Presidents’ Office, Regional Administration and Local Government) and the regional office of Rukwa and its councils.

## Results

Vaccination coverage: at the beginning of the vaccination, the target was 70% of people aged 18 and above must be vaccinated (Table 1).

**Table 1:** total population projection and target population of the number of people eligible for COVID-19 vaccine in Rukwa region by 2021-2022

Target population	Council				
	Sumbawanga Mc	Sumbawanga Dc	Nkasi Dc	Kalambo Dc	Total (Rukwa)
70% of age 18 and above	104,005	135,327	125,479	91,454	456,265
Aged 18 and above	148,579	193,324	179,255	130,649	651,807
**Total population**	407,143	289,002	377,636	277,539	1,351,320

**Figure 1:**
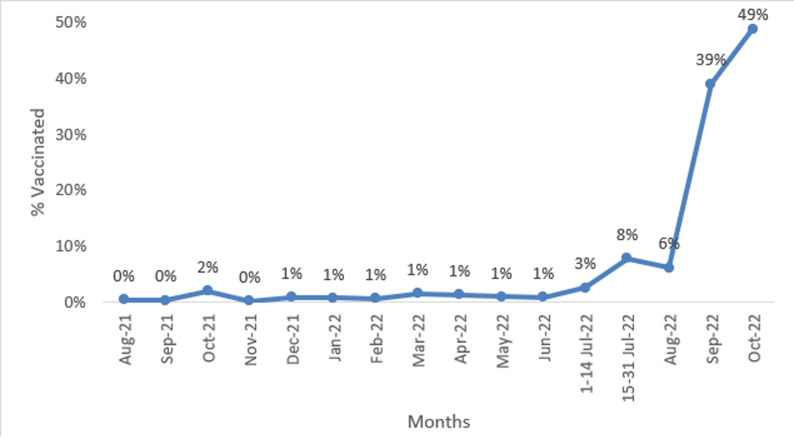
COVID-19 monthly vaccination trend in Rukwa region from August 2021 to October 2022

As COVID-19 vaccination progressed, the provision of the COVID-19 vaccine in Rukwa region started early on August 2021, and the number of people vaccinated was very low. The coverage continued to be low each month until the start of the campaign, which started from 15th July to 31st August 2022. Before the beginning of the campaign, the coverage of the number of people vaccinated was very low (October 2021 to June 2022, (Table 2)). During the campaign, the number of people vaccinated was more than five times compared to any previous months before the start of the campaign. A rapid increase happened during the campaign from 10% to 22% in cumulative percentage since the beginning. A 12% increase in the of people vaccinated was higher than any change before the start of the campaign (Figure 1).

**Table 2:** number of people fully vaccinated from the start of COVID-19 vaccine provision in Rukwa region, August 2021 - October 2022

Months	Sumbawanga Mc	Sumbawanga Dc	Nkasi Dc	Kalambo Dc	Rukwa
Aug-21	193	1,669	501	383	2,746
Sep-21	298	673	955	411	2,337
Oct-21	2,654	2,829	3,260	3,918	12,661
Nov-21	454	659	170	219	1,502
Dec-21	1,588	1,771	1,571	1,089	6,019
Jan-22	1,324	1,093	1,185	1,140	4,742
Feb-22	984	1,209	1,146	1,025	4,364
Mar-22	1,968	1,682	3,365	2,739	9,754
Apr-22	1,349	1,015	3,995	2,608	8,967
May-22	563	516	2,623	2,573	6,275
Jun-22	423	748	2,127	2,077	5,375
July 22	14,230	19,788	17,998	15,889	67,905
Aug-22	12,201	9,543	10,268	8,020	40,032
Sept -22	52,185	69,886	77,931	53,820	253,822
Oct-22	75,001	87,665	83,044	71,844	317,554
**Total**	165,415	200,746	210,139	167,755	744,055

Comparison of coverage against other regions: even after the campaign, Rukwa was still the last region in vaccine coverage percentage by 24%. But at the end of October 2022, Rukwa was among the top five regions in vaccine coverage with the highest percentage increase marginal change of 70% from September to October.

**Figure 2:**
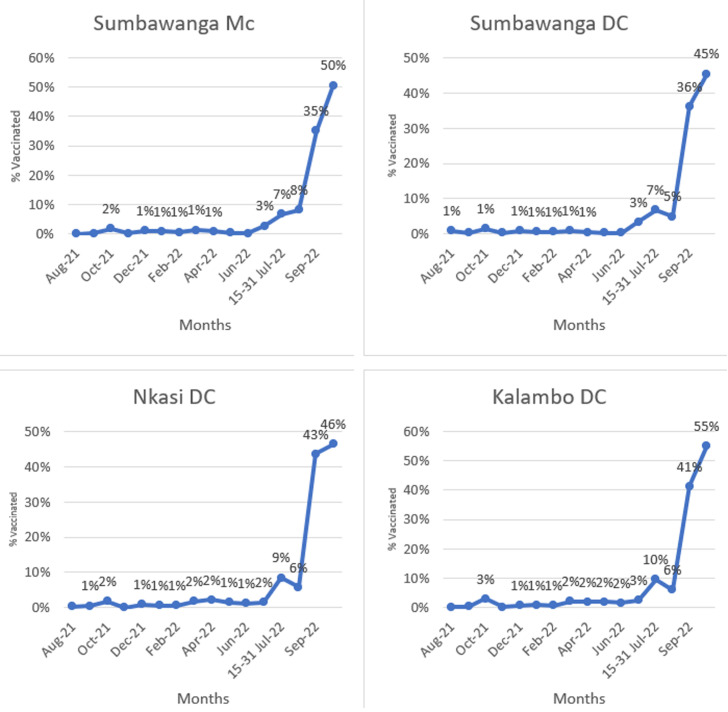
comparison of COVID-19 coverage rate between Rukwa districts from August 2021 to October 2022

Community champions contribution: during and after the implementation of the campaign, community champions used data reporting tools to document their efforts by reporting the number of people they sensitized and influenced to be vaccinated (Figure 2). The data shows that 57% of people sensitized by community champions were vaccinated in Sumbawanga DC and 65% of people sensitized in Nkasi DC. Also, the data showed the percentage of females sensitized and vaccinated in both councils compared with males (Table 3).

**Table 3:** community champions' contribution toward the number of people sensitized against vaccinated

Indicators	Sumbawanga Dc		Nkasi Dc	
	Male	Female	Male	Female
People aged 18 and above sensitised	30,492	39,700	6,899	10,289
People vaccinated	16,621	23,251	4,258	6,851
Percentage of people sensitised and vaccinated	55%	59%	62%	67%

## Discussion

The champion campaign has proven to be effective in contributing to the rise in the number of people who demanded COVID-19 vaccination. This approach is the same as what was used in India to eradicate polio [13,15] refusing to take their children to get polio vaccines. However, the approach used in India encountered a challenge of persistent resistance which led to several visits for the provision of awareness and sensitization compared to the approach used in Tanzania for COVID-19 vaccines. The champions succeeded in changing the behavior of the communities during a single visit because of testimonies that were given by the champions themselves. We found that by using data reporting tools which were used by champions to document their efforts through reporting the number of people sensitized and those who demanded vaccination and later entered the health promotion monitoring and evaluation web-based system for recording and analysis of the effectiveness of the campaign has made it easier to evaluate the efforts and contribution made by champions. This was unlike the community engagement efforts, which were done previously with no documentation. However, more resource mobilization is needed to equip champions with mobile devices and the Internet to enter data directly into the web-based system. We also found that the launching of the campaign which was done with the presence of high-level political leaders has contributed to the majority of interventions being conducted with success. After the launch, the regional and council team and champions began to own the project and engaged more in sensitization and community education to ensure they reached vaccination targets.

Massive gathering meetings were conducted, awareness was conducted in the facility during reproduction, and child health clinics and outpatient departments, sensitization in women groups (locally known as VIKOBA and SACCOS) meetings especially for women champions, sensitization in entertainment areas where people are watching sports, playing grounds and local beer, village meetings gatherings where communities are buying fertilizers, TASAF meetings, and local auctions, sensitized religious leaders were also used as champions, and as educators, this made sensitization in places of worship easy also in religious gatherings It was noted that a single dose of the Johnson and Johnson COVID-19 vaccine was a preferable vaccine by most people and has contributed to the increase in the number of fully vaccinated people.

The limitations of our campaign include religious beliefs among religious leaders who still believe that vaccination has no importance in human life. It was so difficult for champions to convince as it is related to faith someone has. Also, the geographical location in many places led to the inaccessibility of some areas and traveling from one place to another became difficult due to the lack of transport funds and fuel; again, changes in vaccine types were confusing the public, which increased hesitance in decision making. Despite those limitations, the champion campaign was still a success because the improvement in COVID-19 vaccine uptake coverage rate in Rukwa region was notably increased.

## Conclusion

Detailed planning, collecting evidence-based data, continued advocacy, community engagement, and sensitization activities will continue to assist in tackling the number of community health challenges, hesitancy, and other contributing factors hindering the increasing COVID-19 vaccine coverage. Champion intervention contributes to the overall impact and mechanism to improve COVID-19 vaccination coverage. This initiative should be integrated into other campaigns or other disease campaigns that need to use the same approach.

### What is known about this topic

- There is increasing attention on the usefulness of engaging the community in the planning and implementation of immunization activities;
- The involvement of community members in interventions counters inequity, prevailing social norms, mistrust, misinformation, cultural irrelevance and builds ownership and accountability;
- Community champions are most effective when they are trusted members of the community with similar beliefs and characteristics.

### What this study adds

- The use of community champions was an effective strategy in significantly improving COVID-19 vaccination coverage in Rukwa region;
- Endorsement of COVID-19 vaccination by political leadership further improved vaccine acceptance in the community.
